# Motor Activity-Induced White Matter Repair in White Matter Stroke

**DOI:** 10.1523/JNEUROSCI.0631-23.2023

**Published:** 2023-11-29

**Authors:** Miguel A. Marin, Amy J. Gleichman, Xiaofei Wei, Daniel S. Whittaker, Istvan Mody, Christopher S. Colwell, S. Thomas Carmichael

**Affiliations:** ^1^Department of Neurology, David Geffen School of Medicine at UCLA, Los Angeles, California 90095; ^2^Department of Psychiatry and Biobehavioral Sciences, David Geffen School of Medicine at UCLA, Los Angeles, California 90095

**Keywords:** learning, myelin neural repair, rehabilitation, oligodendrocyte, precursor cell

## Abstract

Subcortical white matter stroke (WMS) is a progressive disorder which is demarcated by the formation of small ischemic lesions along white matter tracts in the CNS. As lesions accumulate, patients begin to experience severe motor and cognitive decline. Despite its high rate of incidence in the human population, our understanding of the cause and outcome of WMS is extremely limited. As such, viable therapies for WMS remain to be seen. This study characterizes myelin recovery following stroke and motor learning-based rehabilitation in a mouse model of subcortical WMS. Following WMS, a transient increase in differentiating oligodendrocytes occurs within the peri-infarct in young male adult mice, which is completely abolished in male aged mice. Compound action potential recording demonstrates a decrease in conduction velocity of myelinated axons at the peri-infarct. Animals were then tested on one of three distinct motor learning-based rehabilitation strategies (skilled reach, restricted access to a complex running wheel, and unrestricted access to a complex running wheel) for their capacity to induce repair. These studies determined that unrestricted access to a complex running wheel alone increases the density of differentiating oligodendrocytes in infarcted white matter in young adult male mice, which is abolished in aged male mice. Unrestricted access to a complex running wheel was also able to enhance conduction velocity of myelinated axons at the peri-infarct to a speed comparable to naive controls suggesting functional recovery. However, there was no evidence of motor rehabilitation-induced remyelination or myelin protection.

**SIGNIFICANCE STATEMENT** White matter stroke is a common disease with no medical therapy. A form of motor rehabilitation improves some aspects of white matter repair and recovery.

## Introduction

White matter stroke (WMS) is a vascular disorder that affects deep penetrating blood vessels resulting in ischemic lesions in white matter. The results of WMS are catastrophic, with a large percentage of patients displaying motor and cognitive deficits. WMS stands as a leading cause of vascular dementia (Iadecola, 2013; Alber et al., 2019; Oveisgharan et al., 2022). Pathologic analysis demonstrates that white matter lesions are demarcated by myelin degeneration, axon loss, astrogliosis, and inflammation (Wardlaw, 2008; Bailey et al., 2012; Wardlaw et al., 2015). In aging humans, the incidence of WMS is highly prevalent with up to 100% of people showing evidence of ischemic lesions in white matter by the age of 80 (de Leeuw et al., 2001).

One potential route for therapeutic intervention in WMS may be to regenerate lost and damaged myelin in surviving axons. While the degree of damage to axons within the peri-infarct is severe, ultrastructural analysis indicates that a population of axons remain intact. Therapeutic intervention has been previously demonstrated to enhance remyelination, suggesting that surviving axons are targeted by oligodendrocytes for remyelination (Sozmen et al., 2016, 2019). Myelin serves multiple critical functions, and its loss can have a dramatic impact on the nervous system. One such function is its role in preserving the fidelity of conduction of action potentials by both increasing the membrane resistance and decreasing the capacitance of the axon as well as clustering electrogenic structures called nodes of Ranvier at discrete regions along the axon (Chang et al., 2016). Nodes of Ranvier are found at the 1 µm gaps situated between myelin sheaths and are composed of a dense array of adhesion proteins and cytoskeletal elements that tether voltage-gated ion channels to the axons allowing for saltatory conduction (Kaplan et al., 1997; Rasband, 2004; Rasband and Peles, 2015; Chang et al., 2016). Another critical function is myelin's role in providing metabolic support to the underlying axon, which includes the transfer of glucose and lactate (Lee et al., 2012; Nave and Werner, 2014; Meyer et al., 2018). This would be especially critical in an ischemic environment on which surviving axons would necessitate greater metabolic support from neighboring glial cells, such as oligodendrocytes. The absence of such support would precipitate further axon degeneration (Hinman, 2014).

Previous studies have linked motor activity with enhanced oligodendrocyte progenitor cell (OPC) proliferation, oligodendrogenesis, and *de novo* myelin generation in uninjured mice. Exposure of uninjured mice to either a complex running wheel, or a forelimb reach task enhances oligodendrogenesis and myelin load in the brain (McKenzie et al., 2014; Xiao et al., 2016; Bacmeister et al., 2020). One proposed explanation for these observations is that signaling from neurons onto associated OPCs drives them toward maturation and production of new myelin (Fields, 2015). These motor learning protocols may be adapted to serve as rehabilitation strategies for white matter repair in stroke.

In this study, it was first determined that OPCs respond to WMS in young adult mice by undergoing rapid proliferation and differentiation in the peri-infarct. This effect is completely abolished in aged (18-month-old) mice. WMS decreases the conduction velocity of evoked action potentials at the peri-infarct 30 d after injury. Three motor learning-based rehabilitation strategies (*ad libitum* access to a complex running wheel, 1 h exposure to a complex running wheel, and a forelimb reach task) were tested for their capacity to drive myelin repair. Only *ad libitum* access to a complex running wheel resulted in enhanced oligodendrocyte differentiation. This phenotype is age-dependent. The conduction velocity of evoked action potentials was enhanced to levels comparable to naive controls with this rehabilitative approach, suggesting functional repair following WMS and *ad libitum* complex wheel rehabilitation. Using the NG2creERTM; tau-mGFP reporter mouse line, no changes in remyelination or node of Ranvier reassembly were detected suggesting that *ad libitum* complex wheel does not have an appreciable effect on oligodendrocyte maturation and may work to repair myelinated axon function through another mechanism.

## Materials and Methods

### Mice

All experiments were performed in accordance with National Institutes of Health animal protection guidelines and were approved by the UCLA Animal Research committee. Two- to 3-month-old C57BL/6J mice were obtained from The Jackson Laboratory (strain #000664). Aged 18- to 22-month-old C57BL/6J mice were obtained from the National Institute on Aging, Aged Rodent Colony. NG2-creERTM (The Jackson Laboratory strain #008538) was kindly provided by Harley Kornblum, and the Tau-mGFP reporter mouse was obtained from The Jackson Laboratory (strain #021162). Genotypes were determined by PCR analysis of tail genomic DNA using the appropriate primers (Transnetyx).

### Stroke

WMS was produced as previously described (Hinman et al., 2015; Rosenzweig and Carmichael, 2015; Marin et al., 2023). The vasoconstrictor N5-(1-iminoethyl)-L-ornithine (27 mg/ml in 0.9% saline; EMD Millipore) was injected via micropipette through the cortex and into the white matter ventral to the motor cortex at an angle of 45 degrees (posterior to anterior). Three injections of 200 nl were administered using the following coordinates: (#1: AP 0.75, ML 0.96, DV 2.25; #2: AP 1.00, ML 0.96, DV 2.20; #3: AP 1.25, ML 0.96, DV 2.15). Following stroke, mice were moved to their home cages maintained on a heating pad until the animals recovered from anesthesia.

### Tissue processing for immunofluorescence

Animals were perfused transcardially with cold 1×PBS followed by 4% PFA. Brains were removed and cryoprotected in 30% sucrose. Brains were then flash frozen on dry ice, and 30 µm coronal sections were acquired using a cryostat (Leica CM 0530). Tissue was stored in antifreeze at −2°C. Sections were blocked and permeabilized (5% normal donkey or goat serum; 0.1% Triton X-100) and incubated overnight at 4°C in primary antibody. Sections were then incubated for 90 min in secondary antibody followed by mounting on bovine gelatin-coated slides followed by ethanol dehydration and xylene clarification. Coverslips were mounted with DPX (Millipore Sigma). Slides were imaged using confocal microscopy (Nikon C2) using imaging parameters kept constant across treatment groups. For immunostaining with antibodies raised in mouse, mouse-on-mouse block (Vector Labs) was used before serum blocking to minimize nonspecific labeling. Primary antibodies used in these studies are listed in alphabetical order: mouse IgG2a anti-ankyrin-G N106-36 (Millipore MABN466), rabbit anti-caspr (Abcam 34151), mouse anti-caspr K65/35 (Antibodies 75-001), rabbit anti-Iba1 (Wako 019-19741), goat anti-MOG (Neuromics GT15141), mouse anti-NF-160 (Abcam ab7794), rabbit anti-Olig2 (Millipore AB9610), mouse anti-Olig2 (Millipore MABN50), and goat anti-PDGFRα (Neuromics GT15150). Secondary antibodies used in this study were as follows: AlexaFlour-594 AffiniPure Goat Anti-Mouse IgG Fcy subclass 2a specific, AlexaFluor-647 AffiniPure F(ab′)2 Fragment Goat Anti-Rabbit IgG (H + L) and donkey F(ab)2 conjugated to cy2, cy3, or cy5 (Jackson ImmunoResearch Laboratories).

### Tissue processing for hybridization chain reaction (HCR)

Hybridization and amplification buffer, probes, and hairpins were purchased from Molecular Instruments. Wash buffer was made in-house based on the protocol provided by the company. Animals were killed with an overdose of isoflurane. Brains were immediately removed and flash frozen in dry ice; 10 μm coronal sections were acquired on a cryostat (Leica CM 0530) and mounted on bovine gelatin-coated slides. Labeling for HCR was conducted on slide-mounted sections. In plastic Coplin staining jars, sections were fixed with cold 4% PFA. Slides were transferred to a humidity chamber and incubated in hybridization buffer for 4 h at room temperature. Hybridization buffer was wicked off and replaced with probes mixed in hybridization buffer. Hybrislips (Sigma) were placed atop sections and left to incubate at 37°C for 12-18 h. Hybrislips were removed and sections were washed in Coplin jars in a descending concentration of probe wash buffer (75%, 50%, 20%, and 0%). Sections were moved back to humidity chambers and incubated in amplification buffer for 30 min at room temperature. In parallel, fluorophore-conjugated hairpins were heated to 95°C for 90 s in a thermocycler and allowed to cool for 30 min. Hairpins were then diluted in amplification buffer and added to sections and covered with a Hybrislips for 12-18 h at room temperature. Sections were then moved back to Coplin jars, washed, and stained with DAPI. Sections were dried and run through a dehydration series followed by clarification in xylene. Sections were mounted in DPX for confocal imaging. Probes were custom manufactured by Molecular Instruments and listed in alphabetical order alongside their corresponding accession numbers: BCAS1 (NM_029815.2), ENPP6 (NM_177304.4), and PLP1 (NM_011123.4).

### EdU administration, colabeling with HCR probes, and imaging

EdU labeling destroys HCR signal. An alternative approach was taken to colocalize EdU with HCR probes. EdU was administered as previously described (Marin et al., 2023) Mice were administered EdU (Calbiochem) in their drinking water (200 µg/ml) and given *ad libitum* access through the duration of the experiment. Tissue was then processed for HCR labeling as previously described with an added EdU labeling and imaging step. Briefly, following labeling for HCR and DAPI, coverslips were mounted in a non-hard set aqueous mounting media, and images were immediately acquired with confocal microscopy. Tissue landmarks for imaging location were noted. Following image acquisition, coverslips were washed off in 1× PBS. Tissue was permeabilized (10% Triton X-100) followed by staining for EdU (2 mm sulfo Cy5 azide, 100 mm CuSO_4_, and 500 mm sodium ascorbate). The tissue was restained in 0.02% DAPI, washed in 1× PBS. Images of Edu and DAPI were acquired based on previously noted landmarks and were registered with previously acquired HCR images for analysis.

### Combined ISH/immunohistochemistry

Following ISH and imaging, coverslips were removed and the ISH signal was bleached by submerging the slide in xylene for 3 d at room temperature, not protected from light. After removing the coverslip, residual DPX mounting media was further dissolved by continued xylene incubation for 4 h. Sections were rehydrated in descending concentrations of ethanol (100%, 95%, 70%, 50%), followed by 3 × 5 min washes in PBS. Slides underwent antigen retrieval in 10 mm sodium citrate, pH 6, at 80°C for 30 min. Nonspecific staining was blocked with 5% normal donkey serum and mouse-on-mouse blocking solution (Vector Labs, #MKB-2213) in PBS + 0.3% Triton X-100 for 1 h. Slides were incubated in primary antibody overnight (NF-160: Abcam, ab7794, 1:100) in PBS + 2% NDS + 0.3% Triton X-100. The next day, slides were washed 3 × 5 min in PBS and incubated in secondary antibody (1:500, donkey anti-mouse 561, Jackson ImmunoResearch Laboratories) for 1 h.

### Combined ISH/TUNEL

Slides were processed for ISH with PLP1 and BCAS1 probes as described above. Following ISH processing, slides were processed for TUNEL staining per the manufacturer's protocol (Click-iT Plus TUNEL Assay, Invitrogen/Fisher Scientific, #C01618), beginning with fixation and permeabilization (step 2.1). To minimize background staining, following TUNEL labeling, slides were incubated in TrueView (Vector Labs, #SP-8400) as per the manufacturer's instructions and mounted in Fluoro-Gel (Electron Microscopy Services, #17985-30).

### Compound action potential (CAP)

This approach was adapted from Aguirre et al. (2007) and Sun et al. (2022). Three-month-old male C57B/6J mice were anesthetized with isoflurane before decapitation. Brains were quicky removed, and 400 µm coronal slices containing a stroke were sectioned using a Lecia VT1200S Vibratome in ice-cold N-methyl-D-glutamine-based HEPES-buffered solution (35 mm N-methyl-D-glutamine, 10 mm D-glucose, 4 mm MgCl_2_, 0.5 mm CaCl_2_, 1 mm KCl, 1.2 mm KH_2_PO_4_, 26 mm HEPES, pH 7.4, 290-300 mOsm bubbled with 100% O_2_). Slices were then transferred to an interface chamber in reduced sodium ACSF (85 mm NaCl, 25 mm D-glucose, 55 mm sucrose, 2.5 mm KCl, 1.25 mm NaH_2_PO_4_, 0.5 mm CaCl_2_, 4 mm MgCl_2_, 26 NaHCO_3_, pH 7.3-7.4, bubbled with 95% O_2_, 5% CO_2_) and incubated for 30 min at 36C. Following recovery, the interface chamber was moved to room temperature before recording. Slices were placed in a recording chamber and perfused (2 ml/min) with oxygenated ACSF (126 mm NaCl, 10 mm D-glucose, 2 mm MgCl_2_, 2 mm CaCl_2_, 2.5 mm KCl, 1.25 mm NaH_2_PO_4_, 1.5 mm Na pyruvate, 1 mm L-glutamine, 26 mm NaHCO_3_, pH 7.3-7.4 bubbled with 95% O_2_, 5% CO_2_) at 34°C and visualized with a Nikon SMZ-2B trinocular dissection microscope with attached Nikon Digital Sight 1000 microscope camera. Extracellular field electrodes were filled with 3 mm NaCl, and CAPs were evoked using an electrode connected to a DS3 Isolated Current Stimulator (Digitimer). Amplitude and time duration of evoked potentials were kept constant across all experiments (0.35 mA and 0.10 s). Sections were imaged following recording, and the distance between the recording and stimulation electrode was measured on FIJI. Data were acquired with EVAN (custom-designed LabView-based software). CAPs produced by myelinated (N1) and unmyelinated (N2) axons could be distinguished based on their time duration of the start of the wave relative to the stimulation wave. The investigator was blind to treatment condition (complex running wheel vs wheel lock) during recording and analysis. Peri-infarct was judged subjectively. Recording within the lesion rarely produced detectable signal. Naive controls were used as comparison instead of sham because of concern that the needle placement alone causes damage of the white matter. All salts were purchased from Sigma-Aldrich.

### Rehabilitation

Following WMS, mice were placed back in their home cage and allowed to rest for 5 d. On the fifth day, animals began rehabilitation. Animals were killed 30 d after WMS. For *ad libitum* complex running wheel, animals were single-housed with either a complex running wheel (“Complex wheel”) or with a locked running wheel (“Wheel Lock”). For aged studies, EdU (200 µg/ml) was administered immediately after the stroke. Animals were given *ad libitum* access to this water for the duration of the study. Wheel activity was monitored using an in-cage running wheel system and VitalView Activity Software (Starr Life Sciences). The complex running wheel was custom-designed and manufactured (Peptech) to fit into the running wheel system. This wheel was designed with 36 removeable rungs. Fourteen rungs were removed with gaps no larger than two rungs. The dimensions of the complex wheel are 5 inches in diameter, 5 inches in height, and 2 1/8 inches in width of the wheel.

For restricted access to a complex running wheel, animals were group housed and given access to the complex running wheel 1 h (within the first 2 h of their wake cycle) a day for 5 d a week. Restricted access animals (“1 h CW”) were compared with cohorts subjected to WMS and single housed in a cage without a complex running wheel 1 h per day, 5 d a week (“No Wheel”). Wheel activity was monitored using a custom-built activity monitor. The activity monitor was built on an Arduino Uno Rev3 board and included a data logging shield (Adafruit), PowerBoost 500 Shield with rechargeable battery shield (Adafruit), and prototyping shield with LED feedback (Adafruit). A reed switch sensor and a neodymium ring magnet attached to the outer wall of the complex wheel were used to detect wheel revolutions. Data were logged in 3 min bins. The code for the pedometer was adapted from code designed and generously provided by Harry A. MacKay (Baylor College of Medicine). Animals that did not perform on the complex running wheel were removed from the study. Three mice were removed from this study.

For skilled reach, animals were dual-housed with a clear plastic chamber (Naohiko Okabe, UCLA). Lining the inner walls of the chamber are wells filled daily with 2.5 g of millet seed. The chamber is designed to allow mice to reach for millet seed with their affected forelimb limb. Boxes were replenished each day with a specified amount of millet seed. This task requires no training as these animals are highly motivated to consume millet seeds. As such, animals do not need to be food restricted. Skilled reach cohorts (“Reach”) were compared with animals subjected to WMS and dual housed with an empty chamber (“No Reach”).

### Experimental design and statistical analyses

Newly generated oligodendrocytes were visualized using HCR and were defined as DAPI^+^ cell bodies that showed enriched BCAS1 and ENPP6 signal and colabeled with PLP1. BCAS1 signal is also found outside cell bodies presumably within myelin sheaths. Large 6 × 4 (40×) *z* stacks (1 μm step) of the infarcted white matter were acquired on a Nikon C2 confocal. Quantification was conducted on FIJI. Cells were counted within the infarcted white matter and within the peri-infarct. Infarcted white matter was defined as the white matter excluding the stroke core within the FOV. The peri-infarct was defined as white matter within 100 μm of the edge of the stroke core and was determined using a custom ROI macro generated on FIJI. The stroke core was defined as an area rich in DAPI^+^ cells and devoid of extracellular BCAS1 signal. The distance of each cell was determined using a distance transformation macro designed in FIJI that measures the distance between each cell and the closest edge of the stroke core. The density of cells was calculated by dividing the number of cells within each ROI by its surface area.

Remyelination was measured in the NG2creERTM; tau-mGFP myelin reporter mouse line. Animals were administered subcutaneous tamoxifen (75 mg/kg in corn oil) once a day for 5 d. Upon tamoxifen administration, recombination occurs in NG2 cells, which include OPCs and pericytes. OPCs that transition into tau-positive mature myelinating oligodendrocytes will fluoresce within the cell body and myelin sheaths. Administration of tamoxifen began immediately after WMS to ensure that myelin visualized in tissue was generated after injury. Changes in remyelination were compared between animals subjected to WMS and exposed to *ad libitum* complex running wheel (“Complex Wheel”) and animals not given access to a complex running wheel (“No Rehab”). Remyelination at the peri-infarct was assessed in three ways: measurement of myelin sheath density and length as well as quantification of ratio of myelin sheaths that stained positive to Caspr, a paranodal marker, at their distal end. The latter analysis was defined as paranodal reassembly. All analysis was conducted in FIJI. For the first analysis, 2 × 1 (100×) *z* stacks (0.3 μm step) were taken at the peri-infarct. Individual myelin sheaths were traced using the Neuroanatomy-SNT plugin. From this measurement, myelin sheath density and length were ascertained. To determine paranode reassembly, a separate cohort to tissue was immunostained for Caspr (abcam 34151) and 100× z stacks (0.3 μm step) were taken at the peri-infarct. Analysis was conducted in 200 × 200 μm ROIs set within each stack. GFP and Caspr signal were thresholded, and Caspr and GFP colabeled structures were isolated using the image calculator function within FIJI. The resulting thresholded image was merged as a new channel within the original raw stack to determine whether they met the morphologic criteria for a paranode.

Previously, staining against ankyrinG in the corpus callosum of 30 μm free-floating tissue yielded unreliable results. However, staining tissue from 10 μm on slide tissue yielded clear and reliable results. This approach was used for all studies in node of Ranvier density. Nodes of Ranvier were only counted if they were clearly flanked by a pair of Caspr-labeled paranodes.

All groups were compared with either a paired two-tailed *t* test or a one-way ANOVA with a Tukey's multiple comparison test. Mean values and SDs are shown on all graphs. Statistical significance was set at *p* < 0.05. Analysis was conducted in Prism. Diagrams in this study were generated using BioRender.com.

## Results

### WMS induces significant injury and is accompanied by OPC proliferation and differentiation

**Figure 1.**
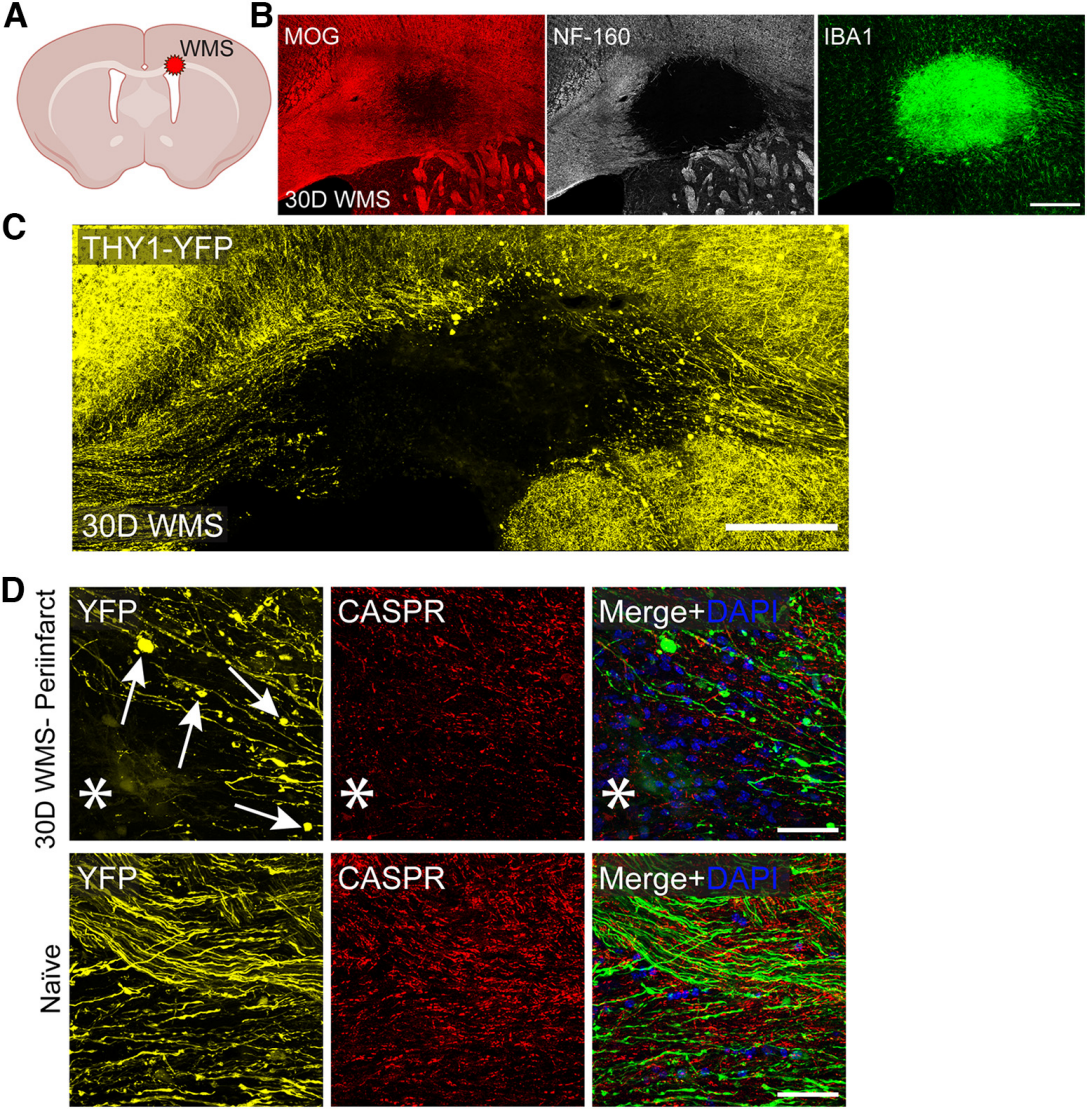
WMS results in axon degeneration, myelin loss, and inflammation. ***A***, Diagram of location of WMS. ***B***, Representative image of infarcted corpus callosum immunostained for myelin (MOG), axons (NF-160), and microglia/macrophages (Iba1) 30 d after WMS. ***C***, ***D***, Corpus callosum of Thy1-YFP mouse immunostained for paranodes (Caspr) 30 d after WMS. Arrows point to axon torpedoes. *Location of stroke. Scale bars: ***B–D***, 200 μm.

Previous studies characterizing the WMS model found considerable tissue degeneration as well as enhanced OPC proliferation within the stroke core and surrounding peri-infarct (Rosenzweig and Carmichael, 2015; Sozmen et al., 2016). This series of studies corroborate previous observations generated in this model. Brain tissue immunostained for markers of myelin (MOG) and axons (NF-160) demonstrated clear degeneration of both structures 30 d after WMS (Fig. 1*B*). This was accompanied by a considerable increase in macrophage/microglia infiltration at the stroke core (Fig. 1*B*). Of note is the presence of diffuse myelin signal along inner boundaries of the stroke core, which shows a clear absence of axons. A closer examination of sparsely labeled axons in the Thy1-YFP mouse shows both a complete loss of axons with the stroke core as well as the formation of retraction bulbs within the peri-infarct, a clear hallmark of axon degeneration (Fig. 1*C*,*D*). Counterstaining with Caspr, a marker of paranodes, demonstrates both a decrease in density as well as disorganization suggesting that myelination, while disrupted, is still present at the peri-infarct (Fig. 1*D*).

**Figure 2.**
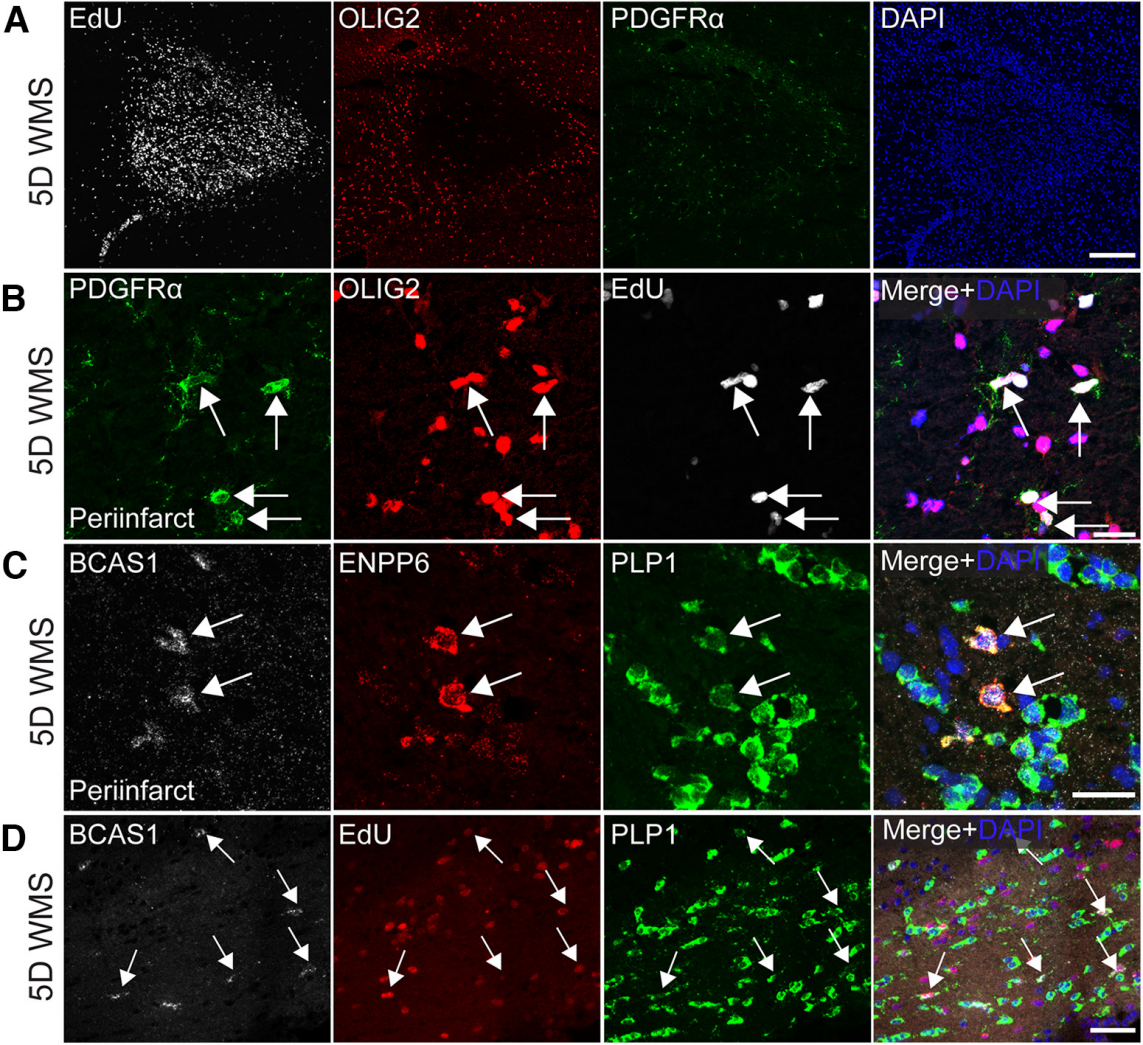
WMS induces OPC and oligodendrocyte differentiation. ***A***, ***B***, Representative images infarcted white matter immunostained against Olig2 and PDGFRα and colabeled for EdU confirms the existence of proliferating OPCs at the peri-infarct (***A***) 5 d after WMS. ***C***, Representative images of infarcted white matter labeled using HCR-FISH for BCAS1, ENPP6, and PLP1 demonstrate the existence of newly differentiated oligodendrocytes at the peri-infarct 5 d after WMS. ***D***, Representative images of newly differentiated oligodendrocytes derived from proliferating OPCs. Scale bars: ***A***, ***B***, 20 μm; ***C***, ***D***, 30 μm.

Analysis of cellular proliferation indicates a rapid mobilization of proliferating cells at the stroke core and peri-infarct 5 d after WMS. (Fig. 2*A*). Colocalization with OPC markers (Olig2 and PDGFRα) to EdU demonstrates that OPCs are undergoing proliferation at the peri-infarct (Fig. 2*B*). To determine whether OPCs undergo differentiation, HCR was used to visualize and track newly differentiated oligodendrocytes. This approach was taken because immunofluorescent signal is sometimes obscured by the presence of the stroke (especially within the peri-infarct), making visualization and quantification of some antibody-visualized markers unreliable. Based on previous studies, newly differentiated oligodendrocytes were defined as cell bodies that demonstrated enriched expression of BCAS1 and ENPP6 and colocalized with PLP1 (Zhang et al., 2014; Xiao et al., 2016; Fard et al., 2017). These studies determined that indeed, a population of differentiating oligodendrocytes can be found at the peri-infarct (Fig. 2*C*). To determine whether newly differentiated oligodendrocytes are derived from proliferating OPCs, EdU was colocalized to cells enriched with BCAS1 and PLP1 (Fig. 2*D*). Nearly 70% of differentiating oligodendrocytes were derived from proliferating OPCs (mean = 69.68253968, SD = 5.681120688).

### WMS induces oligodendrocyte differentiation during the subacute phase of injury in an age-dependent manner

The peri-infarct contains surviving axons adjacent to the stroke core itself but diminished myelination. To determine the time course and extent of response of OPCs to WMS, changes in their differentiation at the peri-infarct (defined as the white matter region up to 100 μm from the edge of the stroke core) as well as the totality of spared white matter (“Ipsilateral white matter”) within the FOV in young adult (2-3 months old) and aged (18 months old) mice was measured. Ipsilateral white matter is defined as subcortical white matter within the ipsilateral hemisphere excluding the stroke core.

**Figure 3.**
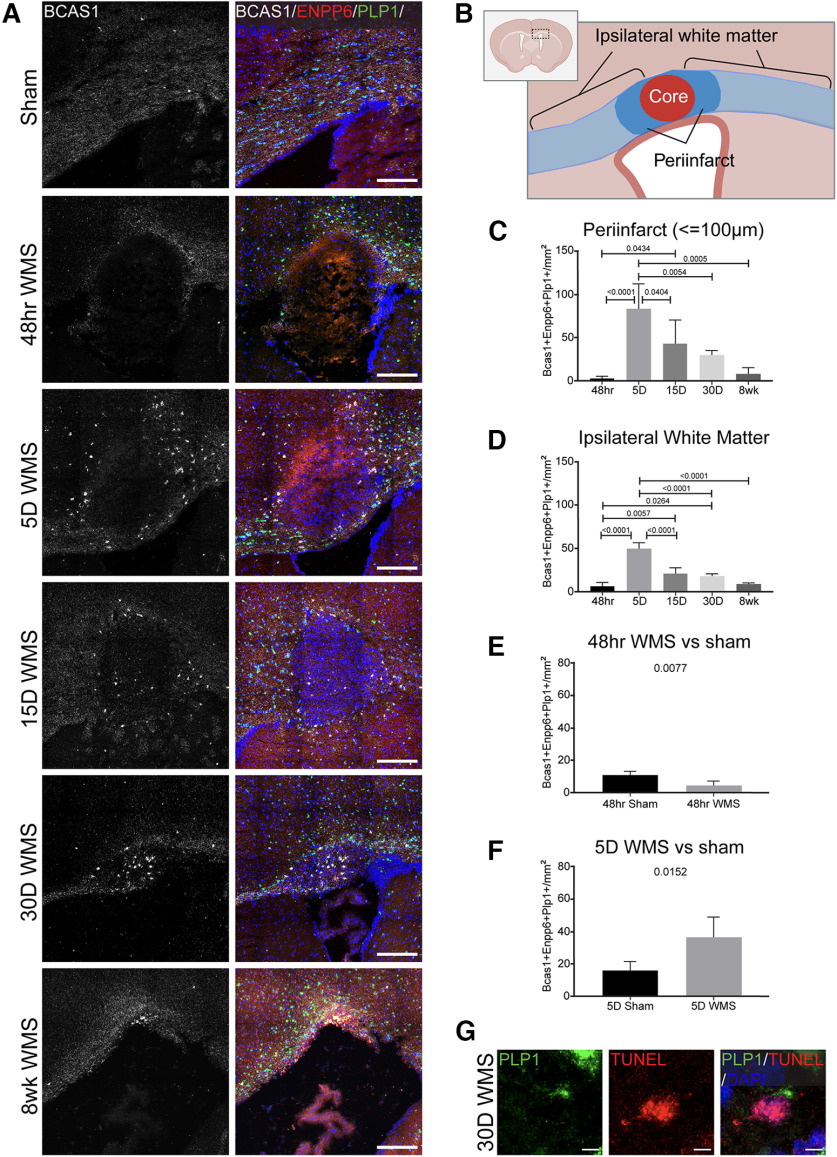
WMS temporarily enhances oligodendrocyte differentiation at the peri-infarct 5 d after WMS in young adult mice. ***A***, Representative images of the progression of oligodendrocyte differentiation following WMS in 2- to 3-month-old mice. ***B***, Diagram of white matter included in analysis: peri-infarct and ipsilateral white matter. ***C***, ***D***, Comparison of the density of newly differentiated oligodendrocytes at the peri-infarct (***C***) and within ipsilateral white matter within the FOV (***D***). ***E***, ***F***, Quantification of the density of newly differentiated oligodendrocytes between 48 h (***E***) and 5 d WMS (***F***) groups and time-matched sham controls. ***G***, Example of PLP^+^ TUNEL^+^ cell in the peri-infarct, 30 d after stroke. Scale bars: ***A***, 200 μm; ***G***, 5 μm.

A population of OPCs was found that undergo rapid differentiation within the subacute phase of the WMS (5 d) within the peri-infarct as well as within the whole of the ipsilateral white matter in young adult mice (Fig. 3*A*,*C*,*D*). The density of newly differentiated oligodendrocytes was significantly increased relative to other time points within the course of the WMS progression (5 d vs 48 h, 15 d, and 30 d WMS) and when compared with time matched sham controls (Fig. 3*C–F*). Oligodendrocyte differentiation persisted 15 and 30 d after WMS relative to the 48 h time point (Fig. 3*C*,*D*). Differentiation was significantly decreased between 48 h WMS and time-matched sham controls, suggesting that either WMS temporarily inhibits differentiation or precipitates early apoptosis of OPCs during the acute phase of infarction (Fig. 3*E*). Previous studies demonstrate increased apoptosis within Olig2^+^ (a pan oligodendrocyte marker) cells 24 and 48 h after WMS in young adult and aged mice, providing support for the later hypothesis (Rosenzweig and Carmichael, 2013). To explore this possibility, we looked for apoptotic oligo lineage cells using TUNEL in combination with BCAS1 and PLP1 HCR across all time points after stroke (48 h, 5 d, 15 d, 30 d, 8 wk). We found no examples of BCAS1^+^ TUNEL^+^ cells; given the relative scarcity of this cell population and the short timing of TUNEL^+^ labeling in the apoptotic process, this is not surprising. However, we did find rare examples of TUNEL^+^ PLP1^+^ cells even as late as 30 d after stroke (Fig. 3*G*). As oligodendrocytes tend to be very long-lived (Tripathi et al., 2017), this may reflect an ongoing stroke-induced response in which OPCs that divide and populate the white matter near the infarct undergo apoptosis at later time points, although a full examination of this process of cell death would likely require a more fine-grained temporal analysis.

**Figure 4.**
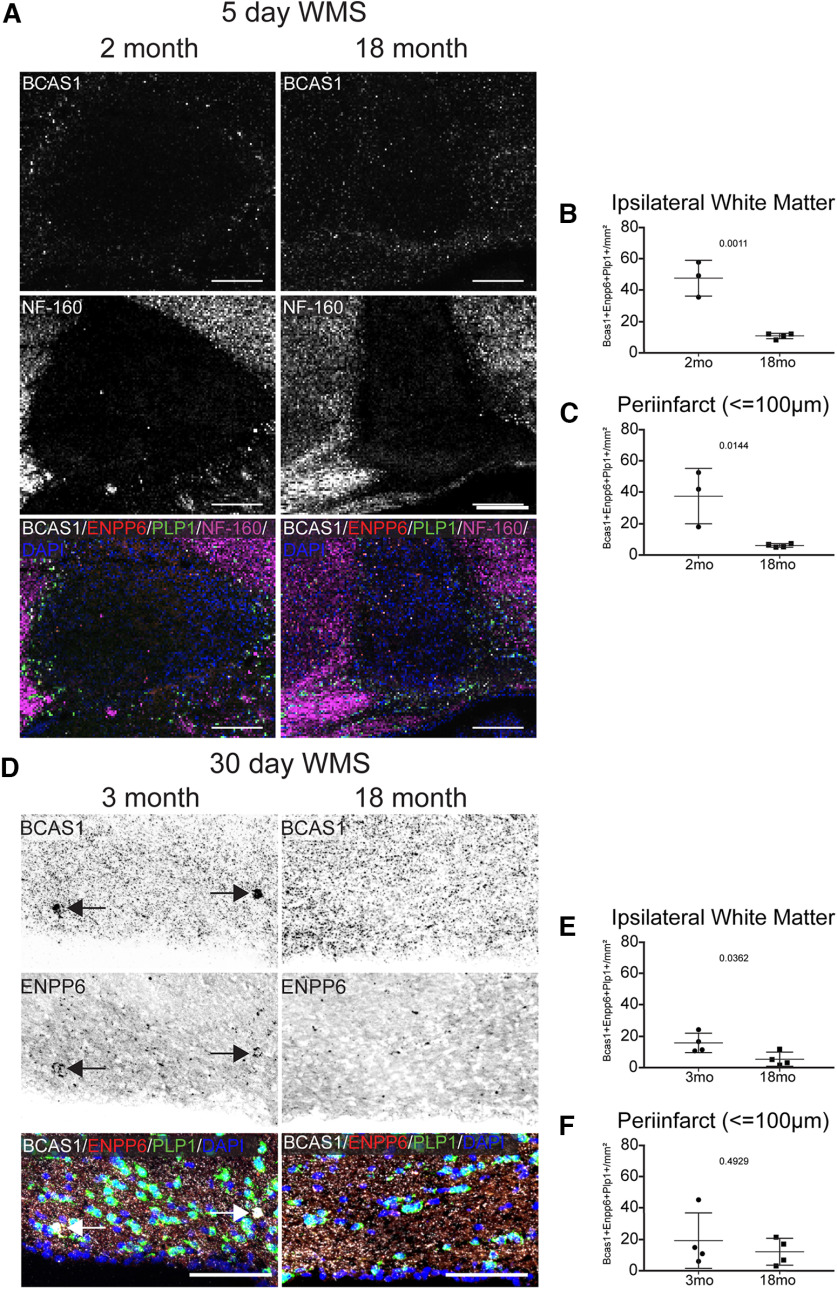
Post-WMS oligodendrocyte differentiation is hindered in aged mice. ***A***, Representative images of oligodendrocyte differentiation (BCAS1^+^) at the peri-infarct 5 d after WMS in 2-month-old and 18-month-old mice, in peri-infarct tissue in which axons are present (NF-160^+^). ***B***, ***C***, Quantification of the density of differentiating oligodendrocytes in ipsilateral white matter (***B***) and the peri-infarct (***C***) 5 d after WMS. ***D***, Representative images of oligodendrocyte differentiation in ipsilateral white matter 30 d after WMS in 3- and 18-month-old mice. ***E***, ***F***, Quantification of the density of newly differentiated oligodendrocytes in ipsilateral white matter (***E***) and within the peri-infarct (***F***) 30 d after WMS. Scale bars: ***A***, 200 μm; ***D***, 100 μm.

To determine whether age impacts WMS induced oligodendrocyte differentiation, OPC response to WMS was assessed in 18-month-old mice and compared with 2- to 3-month-old mice (Fig. 4). OPC differentiation is nearly abolished within the ipsilateral white matter 5 and 30 d after WMS in aged mice (Fig. 4*A*,*B*,*D*,*E*); these are areas in which axons are present (Fig. 4*A*). However, this deficit is found only in the peri-infarct at the 5 d time point in aged mice (Fig. 4*C*,*F*).

These data suggest that a population of OPCs undergo rapid differentiation within a short window of time after WMS. That window follows the end of ischemia-induced apoptosis of the cellular population local to the stroke penumbra and at a time in which OPCs have been documented to undergo proliferation (Rosenzweig and Carmichael, 2013; Sozmen et al., 2016). Some of these OPCs, either mitotic or nonmitotic, will undergo differentiation while others will maintain their progenitor status. This process is age-dependent, with oligodendrocyte differentiation being nearly abolished by 18 months of age.

### Complex running wheel motor rehabilitation enhances oligodendrocyte differentiation in young adult mice following WMS

Previous studies have demonstrated that motor learning enhances oligodendrocyte differentiation and maturation in the uninjured mouse brain (Sampaio-Baptista et al., 2013; McKenzie et al., 2014; Bacmeister et al., 2020). The rationale of the present studies is that these motor learning tasks can be adapted to drive rehabilitation and white matter repair in the WMS model. To achieve this, three motor rehabilitation tasks were evaluated for their ability to induce oligodendrocyte differentiation in the infarcted white mater. Briefly, 5 d after WMS, 2- to 3-month-old mice were subjected to 1 of 3 rehabilitation tasks (*ad libitum* access to a complex running wheel, 1 h access to a complex running wheel, and skilled reach) and killed 30 d after WMS.

**Figure 5.**
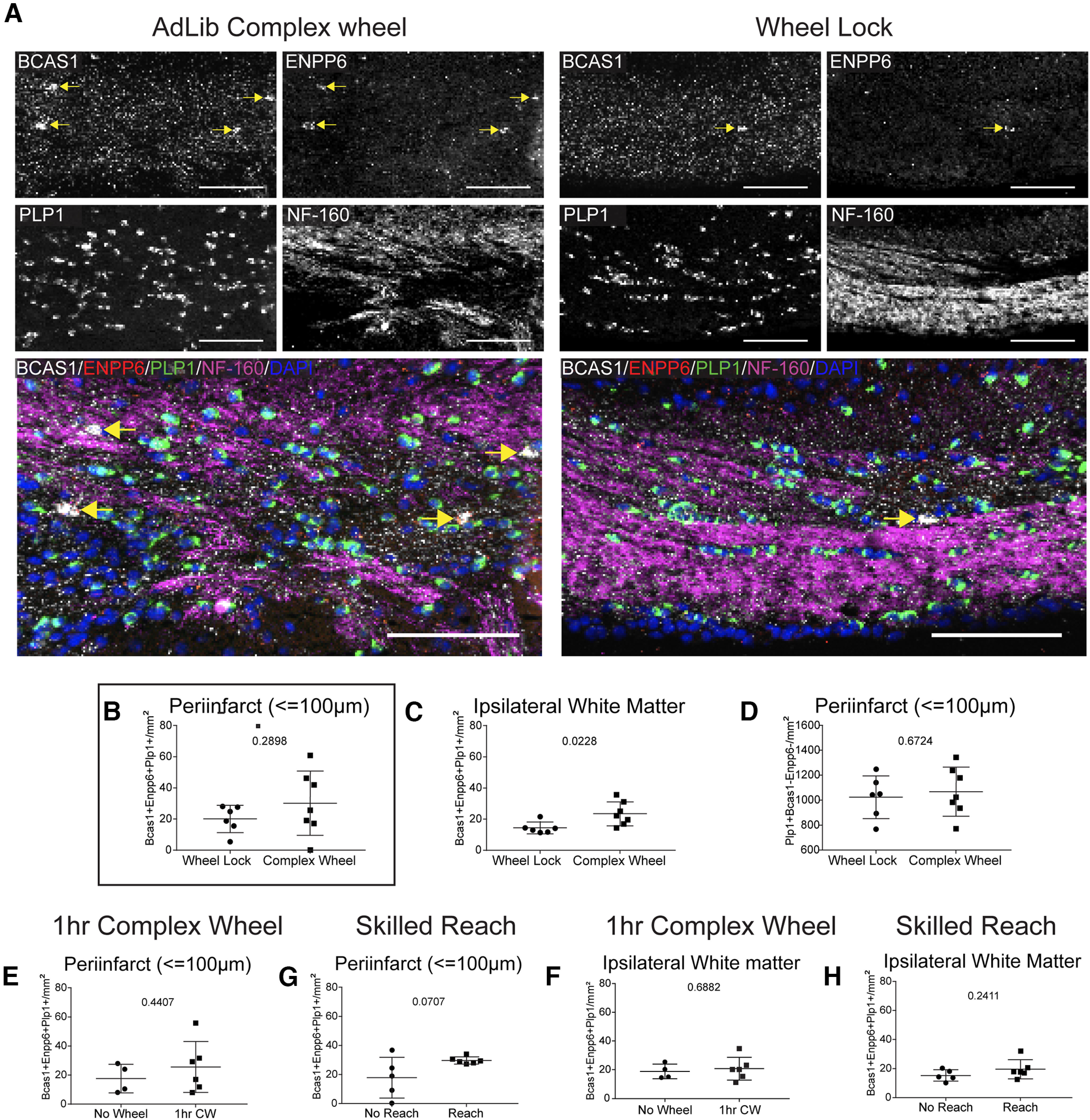
*Ad libitum* access to a complex running wheel alone enhances oligodendrocyte differentiation in ipsilateral white matter. ***A***, Representative images of oligodendrocyte differentiation in ipsilateral white matter following *ad libitum* complex running wheel motor rehabilitation. BCAS1^+^ ENPP6^+^ PLP1^+^ cells are found in axon-containing white matter adjacent to the infarct core (NF-160^+^). ***B***, ***C***, Quantification of the density of differentiating oligodendrocytes within the peri-infarct (***B***) and ipsilateral white matter (***C***) following *ad libitum* complex running wheel motor rehabilitation. ***D***, Quantification of the density of mature oligodendrocytes at the peri-infarct following *ad libitum* complex running wheel motor rehabilitation. ***E***, ***F***, Quantification of the density of differentiating oligodendrocytes within the peri-infarct (***E***) and ipsilateral white matter (***F***) following 1 h complex running wheel motor rehabilitation. ***G***, ***H***, Quantification of the density of differentiating oligodendrocytes within the peri-infarct (***G***) and ipsilateral white matter (***H***) following skilled reach motor rehabilitation. Scale bars: ***A***, 100 μm.

In these studies, the density of newly differentiated oligodendrocytes (BCAS1^+^, ENPP6^+^, PLP1^+^) in infarcted white matter and within the peri-infarct (≤100 μm) was assessed (Fig. 5). Of the three tasks tested, only *ad libitum* complex wheel induced significant changes in oligodendrocyte differentiation in ipsilateral white matter (Fig. 5*C*). Interestingly, this effect was not detected in the peri-infarct, suggesting that enhanced oligodendrocyte differentiation with running wheel activity is not specific to the site of injury but rather is more globally expressed across the white matter (Fig. 5*B–H*). However, there was no change between groups (data not shown) in the more distant subcortical white matter, contralateral to the stroke site. Enhanced neuronal activity can affect the overlying motor cortex and its downstream projections (Gibson et al., 2014). However, there is no significant change in differentiating oligodendrocytes in ipsilateral or contralateral motor cortex or striatum (data not shown). Finally, an analysis of “mature” oligodendrocytes (PLP1^+^, BCAS1^–^, Enpp6^–^) was conducted at the peri-infarct but, no effect was detected (Fig. 5*D*).

### Complex running wheel motor rehabilitation induces functional repair in young adult mice following WMS

**Figure 6.**
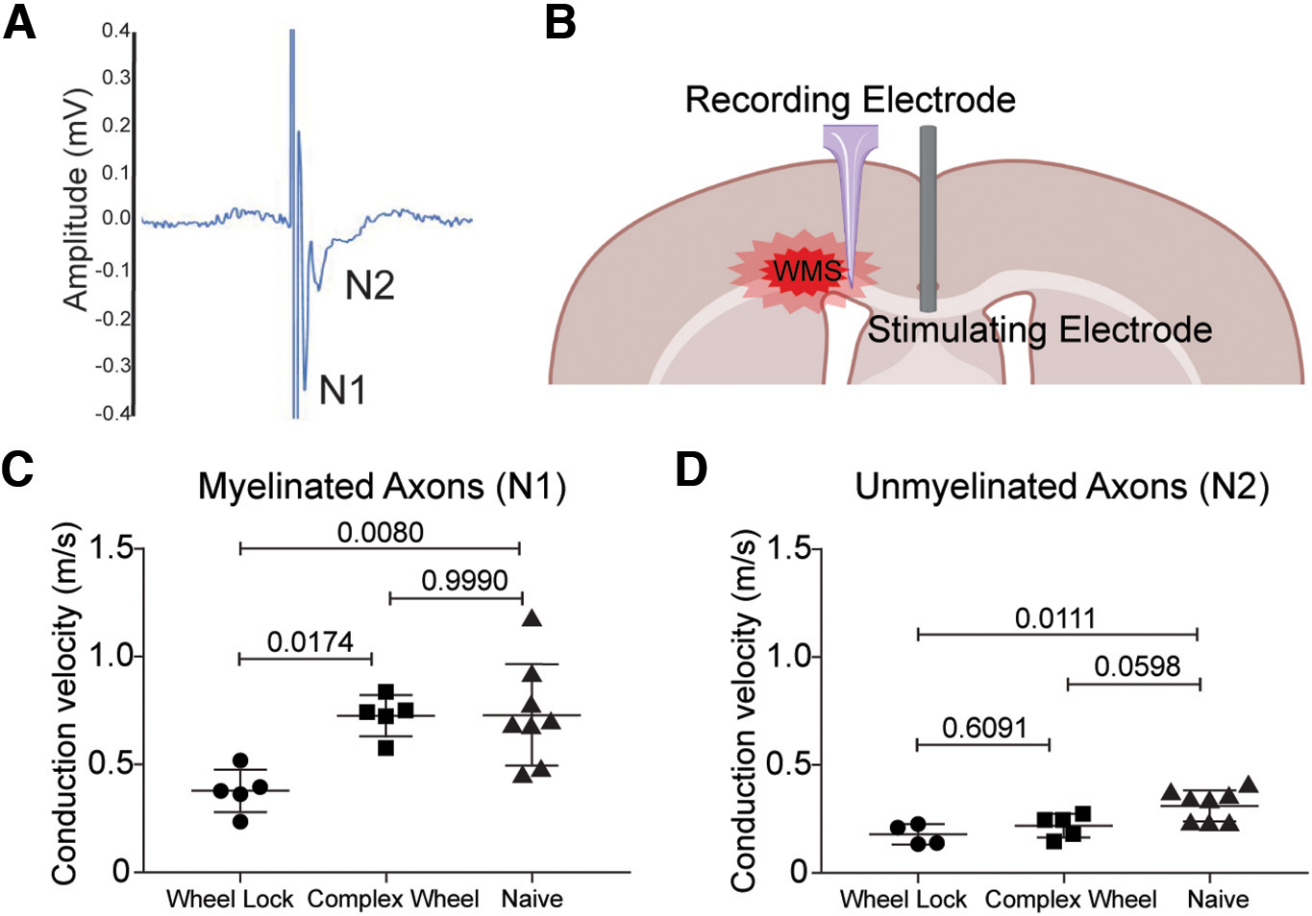
*Ad libitum* access to a complex running wheel promotes functional recovery of myelinated axons after WMS by enhancing conduction velocity speed of CAP. ***A***, Representative trace of a CAP demonstrating signal from myelinated (N1) and unmyelinated (N2) axons. ***B***, Diagram of recording setup. ***C***, ***D***, Quantification of conduction velocity of myelinated axons (***C***, N1) and unmyelinated axons (***D***, N2) CAPs between no rehabilitation stroke controls (wheel lock), *ad libitum* complex wheel (complex wheel), and naive animals.

The discovery that *ad libitum* complex running wheel enhances oligodendrocyte differentiation in infarcted white matter suggests that possibility of functional recovery. Previous published and unpublished attempts to ascertain motor deficits in this WMS model in young adult mice have yet to yield measurable differences between injured and uninjured controls (Rosenzweig and Carmichael, 2013). To circumvent this limitation, conduction velocity of CAPs was measured at the peri-infarct in WMS cohorts rehabilitated with *ad libitum* complex running (“Complex Wheel”; Fig. 6), in myelinated (N1) and unmyelinated axons (N2) (Fig. 6*A*). Both wave forms were distinguished based on their time duration relative to the stimulus artifact. A stimulating electrode was placed at the midline corpus callosum and CAPs were recorded by placing a recording electrode at the peri-infarct (Fig. 6*B*). Complex wheel cohorts were compared with animals subjected to WMS and single housed with a locked running (“Wheel Lock”) and uninjured controls (“Naive”). WMS alone resulted in a significant decrease in conduction velocity of action potentials from myelinated and unmyelinated axons relative to naive controls (Fig. 6*C*). Exposure to *ad libitum* complex wheel significantly increased the conduction velocity of N1 signal relative to wheel lock, which was comparable to naive cohorts suggesting rescue of function of myelinated axons (Fig. 6*C*,*D*).

### Complex running wheel motor rehabilitation does not induce remyelination in young adult mice following WMS

**Figure 7.**
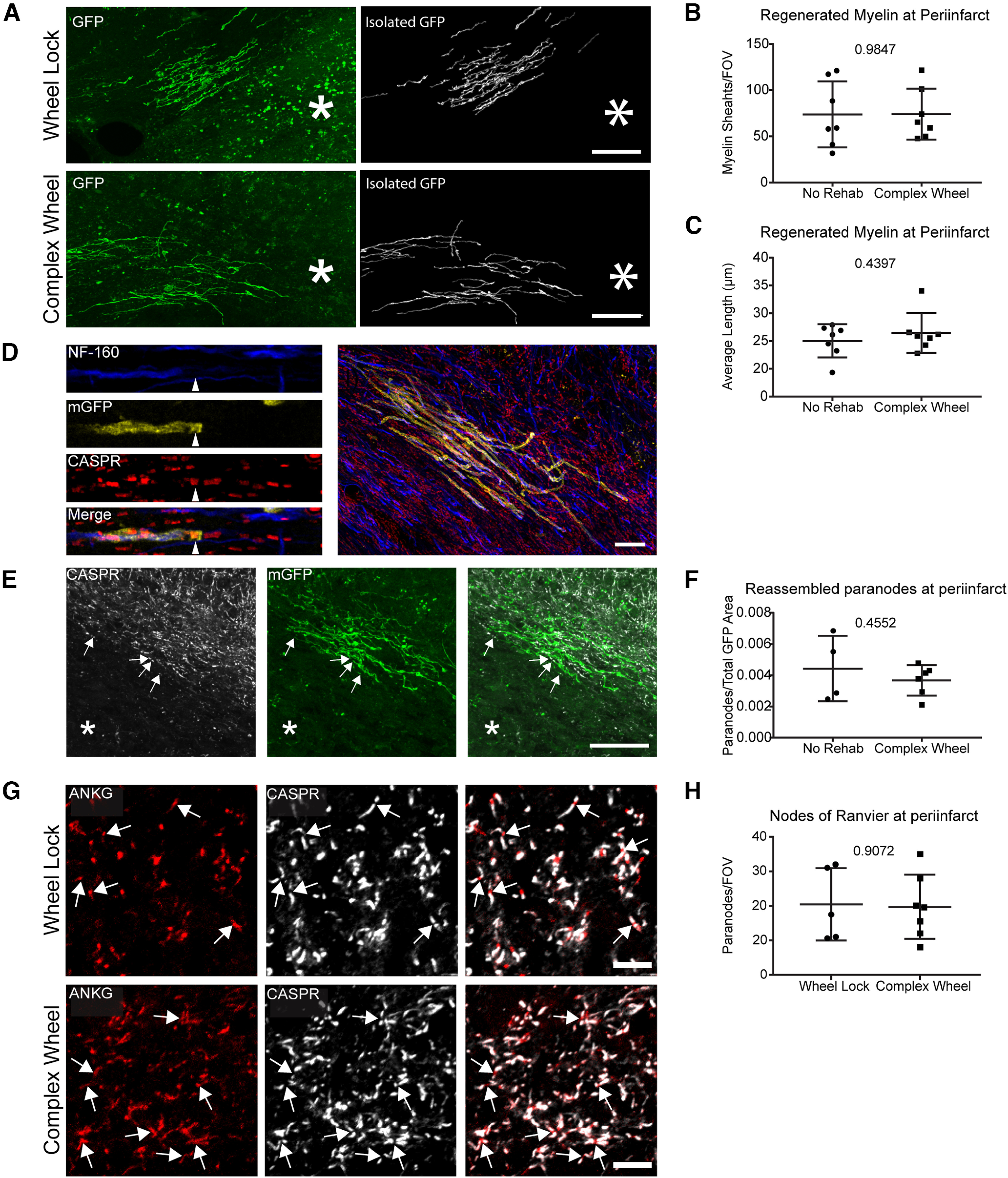
*Ad libitum* access to a complex running wheel does not enhance remyelination, nor does it enhance recovery of nodes of Ranvier at the peri-infarct following WMS. ***A***, Representative images of regenerated myelin formed at peri-infarct and the same myelin sheaths isolated for quantification. ***B***, ***C***, Quantification of the number of myelin sheaths per FOV at the peri-infarct (***B***) and their average length (***C***). ***D***, Representative images of a paranode colocalized to myelin sheaths in subcortical white matter. ***E***, Representative images of paranodes colocalized to myelin sheaths at the peri-infarct. ***F***, Quantification of the ratio of paranodes colocalized to regenerated myelin sheaths to the total area of GFP at the peri-infarct. ***G***, Representative images of the nodes of Ranvier at the peri-infarct. ***H***, Quantification of the number of nodes of Ranvier per FOV at the peri-infarct. *Location of stroke. Scale bars: ***A***, ***D***, ***E***, 40 μm; ***G***, 10 μm.

Enhanced functional recovery in white matter after motor rehabilitation could be explained by differentiating oligodendrocytes maturing and forming *de novo* myelin sheaths on spared axons within the peri-infarct. While complex wheel running increased the number of differentiating oligodendrocytes (BCAS1^+^, ENPP6^+^, PLP1^+^, Fig. 5*C*), but not the overall number of mature oligodendrocytes (PLP1^+^, BCAS1^–^, ENPP6^–^, Fig. 5*D*), this cellular analysis does not reflect myelination itself. Analysis of myelin sheaths generated after WMS and during rehabilitation in the NG2creERTM;tau-mGFP myelin reporter line indicates no difference between conditions using multiple measures (Fig. 7). These include no significant differences in sheath density and average length (Fig. 7*B*,*C*) and number of mature oligodendrocytes (data not shown). A separate cohort of tissue was then stained against Caspr, a paranodal marker, and colocalized to the distal ends of GFP^+^ myelin sheaths (Fig. 7*D*,*E*), which will visualize paranodal architecture, an addition to the myelin architectural studies. There are no differences between cohorts (Fig. 7*F*).

Additional studies characterized myelin protection, as opposed to remyelination. Tissue from animals exposed to the *ad libitum* complex running wheel (“Complex Wheel”) were stained with markers of nodes of Ranvier (Ankyrin) and paranodes (Caspr) and their density determined at the peri-infarct. Nodes of Ranvier are excitable axon subdomains, which are found in myelinated axons and are necessary for the saltatory conduction (Chang and Rasband, 2013). Because there is no indication of remyelination, any changes in the density of nodes of Ranvier with motor-learning rehabilitation could be because of enhanced survival following WMS and could explain the CAP results. There is no difference between the motor-learning activity paradigm of *ad libitum* complex wheel and control stroke (Fig. 7*G*,*H*).

These results, coupled with the previous analysis of mature oligodendrocyte density (PLP1^+^, ENPP6^–^, BCAS1^–^) within the peri-infarct after rehabilitation (Fig. 5*D*), suggest that functional recovery is not mediated by the maturation of newly differentiated oligodendrocytes into myelinating oligodendrocytes or the preservation of existing myelin sheaths.

### Complex running wheel motor rehabilitation does not enhance oligodendrocyte differentiation in aged mice following WMS

Thus far, these studies have demonstrated that exposure of the *ad libitum* complex wheel following WMS increases oligodendrocyte differentiation and conduction velocity of action potentials from myelinated axons at the peri-infarct. The next series of studies attempted to replicate some of these findings in aged mice. WMS is a disease commonly associated with age, and determining the efficacy of this rehabilitation approach in 19- to 22-month-old mice would be clinically relevant. As previously noted (Fig. 4), it is at this age that WMS-induced oligodendrocyte differentiation is inhibited in aged mice compared with young adults after WMS.

**Figure 8.**
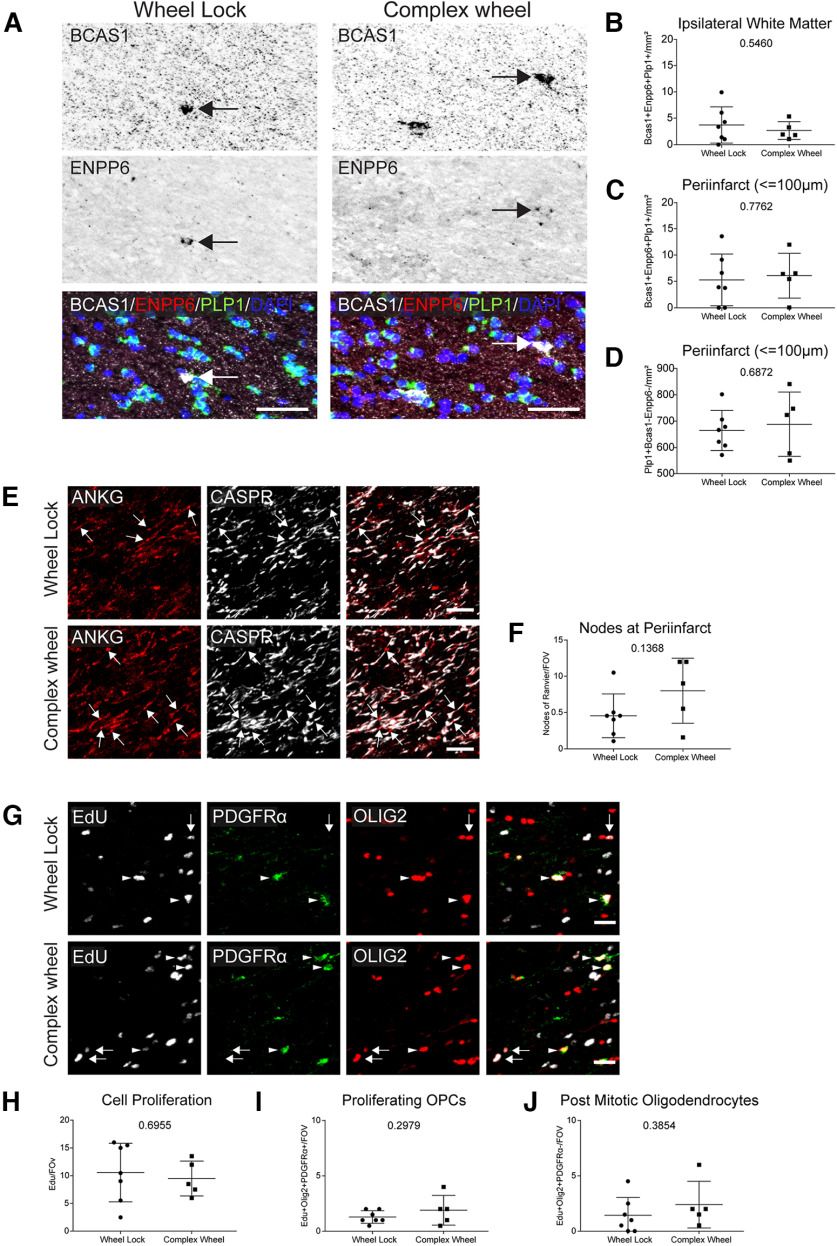
*Ad libitum* access to a complex running wheel does not enhance oligodendrocyte differentiation in aged mice. ***A***, Representative images of oligodendrocyte differentiation in ipsilateral white matter following *ad libitum* complex running wheel motor rehabilitation. ***B***, ***C***, Quantification of the density of differentiating oligodendrocytes within the peri-infarct (***B***) and ipsilateral white matter (***C***) following *ad libitum* complex running wheel motor rehabilitation. ***D***, Quantification of the density of mature oligodendrocytes at the peri-infarct following *ad libitum* complex running wheel motor rehabilitation. ***E***, Representative images of the nodes of Ranvier at the peri-infarct following *ad libitum* complex wheel motor rehabilitation. ***F***, Quantification of the number of nodes of Ranvier per FOV (arrows) at the peri-infarct. ***G***, Representative images of proliferating cells, proliferating OPCs (arrow heads), and postmitotic oligodendrocytes (arrows) at the peri-infarct following *ad libitum* complex running wheel motor rehabilitation. ***H***–***J***, Quantification of the number of proliferating cells (***H***), proliferating OPCs (***I***), and postmitotic oligodendrocytes (***J***) per FOV. Scale bars: ***A***, 50 μm; ***E***, 10 μm; ***G***, 20 μm.

Exposure to the complex wheel does not alter OPC proliferation or maturation, nor does it increase myelination in infarcted white matter (Fig. 8). There was no change in the density of differentiating oligodendrocytes (BCAS1^+^, ENPP6^+^, PLP1^+^) either in the infarcted white matter or within the peri-infarct (≤100 μm) (Fig. 8*A*,*B*). There was also no change in the density of mature oligodendrocytes (PLP1^+^, BCAS1^–^, ENPP6^–^) within the peri-infarct (≤100 μm) (Fig. 8*D*). Tissue was stained for markers of nodes of Ranvier (AnkyrinG) and paranodes (Caspr), and no significant change in their density within the peri-infarct was found (Fig. 8*E*). Finally, tissue was stained for EdU and colocalized with markers for oligodendrocytes (Olig2^+^, PDGFRα^+^) and postmitotic oligodendrocytes (Olig2^+^, PDGFRα^–^). No changes in the density of proliferating cells (Edu^+^), proliferating OPCs, or postmitotic oligodendrocytes was found (Fig. 8*G–J*). However, cell proliferation (Edu^+^) was significantly increased in the dentate gyrus as was neurogenesis (Edu^+^ NeuN^+^) in the subgranular zone and oligodendrogenesis (Edu^+^, Olig2^+^, PDGFRα^–^) in the hilus (data not shown). These data suggest that complex running wheel exposure does indeed have a physiological impact on the aged brain and lends credence to the efficacy of the running wheel model. However, its impact on myelin repair after WMS in the aged brain is not supported.

## Discussion

White matter ischemia and small strokes accumulate to form vascular dementia, which leads to cognitive and motor deficits (Iadecola, 2013; Alber et al., 2019; Oveisgharan et al., 2022). Degeneration of myelin and disorganization of axonal structure normally organized by myelin sheaths serves as a primary pathology in vascular white matter lesions and adjacent white matter (Iadecola, 2013; Hinman et al., 2015; Coelho et al., 2018). Thus, strategies that target myelin repair at or near the site of infarction are critical. Previous studies in a mouse model of WMS have identified OPCs, a progenitor population that gives rise to mature myelinating oligodendrocytes, as a possible source of new myelin at the peri-infarct, the region adjacent to the infarct core which is demarcated by the presence of spared hypomyelinated axons. This study determined the response of OPCs to WMS, how aging affects this process, and the capacity of OPCs to differentiate and mature into myelinating oligodendrocytes after motor rehabilitation.

OPCs undergo rapid differentiation during the subacute phase of WMS in young adult mice. Differentiating oligodendrocytes represent a sparse cell population in naive white matter. However, after WMS, their population density rises significantly at the peri-infarct and adjacent white matter. This effect is most pronounced 5 d after infarction, and steadily decreases at the chronic stage of injury. This process is age-dependent, with a near total loss of differentiating oligodendrocytes within the infarcted white matter in 18-month-old mice 5 and 30 d following WMS. These results corroborate previous studies of myelination in aged mice after WMS, which demonstrated increased oligodendrocyte apoptosis and increased white matter atrophy in aged mice relative to young adult mice (Rosenzweig and Carmichael, 2013). Finally, it was also determined in this study that CAP conduction velocity is significantly decreased at the peri-infarct: a site composed of degenerated and surviving axons. This indicates a functional deficit in axonal conduction in this region. These data indicate that, while the young adult brain maintains some capacity for self-repair in the guise of oligodendrocyte differentiation, functional deficiency is still apparent.

Rehabilitation after stroke and in vascular dementia remains the main therapeutic modality for recovery or the amelioration of deficits clinically. In preclinical studies, motor activity and motor learning provide a strong signal in axo-glial communication that mediates changes in myelination, with increased oligodendrocyte differentiation and increased myelination. Neurorehabilitation in stroke has focused on general aerobic exercise (Gordon et al., 2013) and on specific engagement of the affected limb, such as in reaching and task-specific activity (Dromerick et al., 2021). The present study explored both of these modalities to determine whether increased motor activity, modeled on neurorehabilitation approaches that are used clinically, could induce changes in oligodendrocyte function and enhance remyelination in damaged white matter. Of the three tasks, *ad libitum* activity on a complex running wheel significantly enhanced oligodendrocyte differentiation in white matter adjacent to the infarct in an age-dependent manner. This is an intervention that produces general cardiovascular exercise and motor learning. Interestingly, task-specific neurorehabilitation, in which the mouse repeatedly reaches for a food item up to hundreds of times a day, did not enhance a measure of myelin repair. It was then determined that this rehabilitation strategy of *ad libitum* running wheel activity increases conduction velocity of CAPs at the peri-infarct suggesting functional repair. Thus, in young adult mice, WMS damages the propagation of signals in the injured white matter adjacent to the lesion, and this is restored by one form of rehabilitative therapy. The form of rehabilitative activity that was effective in promoting oligodendrocyte differentiation in young adult mice after WMS was not effective in aged mice. This age effect may be because of differences in this OPC progenitor pool with aging, including in the proliferation and differentiation rate of OPCs (Nishiyama et al., 2021), altered chromatin structure or epigenetic control (Tiane et al., 2019), and different infarct structure (stiffer) (Segel et al., 2019). Does this restoration in white matter function with rehabilitative activity in young adults occur via remyelination?

Remyelination was assessed by visualizing *de novo* myelination in the peri-infarct using the NG2creERTM;tau-mGFP myelin reporter line, a modified approach that fully labels newly born oligodendrocytes (Young et al., 2013) and provides for high-resolution imaging of newly formed oligodendrocytes and their myelin sheaths in normal, stroke, and stroke plus rehabilitation brains. These studies found no evidence of remyelination with the form of rehabilitation that restores axonal conduction in injured white matter. To determine whether rehabilitation induced myelin protection, rather than myelin repair, nodes of Ranvier and mature oligodendrocyte density were measured at the peri-infarct. However, no effect of rehabilitation was seen, suggesting that the formation or maintenance of myelination may not be central to functional repair.

These studies on motor rehabilitation and its effect on oligodendrocyte differentiation are in line with previous studies of myelin plasticity, which demonstrate that learning and neural activity directly impact oligodendrocyte maturation. Motor learning in naive mice increases oligodendrogenesis in subcortical white matter and in the motor cortex. Exposure to a complex running wheel or a forelimb reach task increases oligodendrogenesis (McKenzie et al., 2014; Xiao et al., 2016; Bacmeister et al., 2020), while direct stimulation of neural activity enhances oligodendrocyte development both *in vitro* and *in vivo* (Stevens et al., 2002; Gibson et al., 2014). Interestingly, in these present studies, we found evidence that the complex running wheel, a reported motor learning task, is indeed able to enhance oligodendrocyte differentiation. However, despite evidence of functional repair, there is no evidence for remyelination suggesting that the mechanism of action is not the maturation of oligodendrocytes and opens the possibility of alternative explanations.

One possibility for the effect of rehabilitative activity on the post-stroke white matter is that newly differentiating oligodendrocytes serve alternative functions other than as a transitional phase between progenitor and mature states. In other words, OPCs may promote neural repair after WMS in a process that does not involve a canonical progression to cell maturity to newly generated oligodendrocytes. These alternative neural repair processes might involve soluble factors, cell–cell communication mechanisms, or neuroinflammation. OPCs engage in neuromodulation through soluble factors, such as NG2 ectodomains (Sakry and Trotter, 2016), which regulate synaptic plasticity and glutamatergic signaling. The therapeutic effect of OPC transplantation on white matter injury in spinal cord disease has been attributed in part to secreted growth factors, such as BDNF (Zhang et al., 2006; Manley et al., 2017). Wnt signaling in OPCs regulates synaptogenesis and neuronal extracellular matrix structures, directly affecting circuit function through the secreted factor Wnt Inhibitory factor 1 (Yu et al., 2022). OPCs may also influence white matter damage and repair through cell–cell communication. OPCs alter axonal structure and collateralization during development, and through phagocytosis, axonal elimination (Buchanan et al., 2022). OPCs mediate some aspects of activity-dependent synaptic refinement and remodeling of a connectional network (Auguste et al., 2022), which may have some theoretical relationship to the activity-dependent effect on axonal properties in this present study. Disease states can trigger a phenotypic shift into a pro-inflammatory state for OPCs (Akay et al., 2021). Among the actions in this state, OPCs release cytokines and chemokines that communicate to microglia and other cells, and alter disease pathology. One interesting pathway from the perspective of neural repair in stroke is the OPC release of CCL5 (Moyon et al., 2015), whose receptor mediates a multilevel neural repair pathway in neurons after stroke (Joy et al., 2019).

In conclusion, these studies describe the effect of WMS on OPC dynamics, and the effect motor rehabilitation has on repair, pointing toward motor effects on myelin repair and tissue responses that are delinked from a mechanism of progressive differentiation and myelination within the oligodendrocyte lineage. Determining the molecular mechanism by which activity drives oligodendrocyte differentiation and the role of these differentiating cells may prove critical toward the development of translatable strategies for white matter repair.
